# Mechanical ventilation induces lung and brain injury through ATP production, P2Y1 receptor activation and dopamine release

**DOI:** 10.1080/21655979.2021.2022269

**Published:** 2022-01-16

**Authors:** Wei Wei, Zhentao Sun, Shifeng He, Wanyue Zhang, Sai Chen, Ya-Nan Cao, Ning Wang

**Affiliations:** Department of Anesthesiology, Pain and Perioperative Medicine, The First Affiliated Hospital of Zhengzhou University, Zhengzhou, Henan, China

**Keywords:** Mechanical ventilation, lung-brain interaction, ATP, P2Y1R, dopamine, hippocampus, cognitive dysfunction, bronchoalveolar lavage fluid

## Abstract

Mechanical ventilation can induce lung injury and exacerbate brain injury due to lung-brain interaction. The current study sought to investigate the mechanism of lung-brain interaction induced by mechanical ventilation and offer theoretical insight into the management of ventilator-induced brain injury. The experimental mice were assigned into the spontaneously breathing group and the mechanical ventilation group and injected with dopamine (DA) receptor antagonist haloperidol or P2Y1 receptor antagonist MRS2279 before ventilation. *In vitro* assay was conducted using lung epithelial cells MLE-12 hippocampal neuron cells and HT-22. Mouse recognition function and lung injury were examined. The condition and concentration of neurons in the hippocampus were observed. The levels of several inflammatory factors, DA, adenosine triphosphate (ATP), P2Y1R, and dysbindin-1 were detected. Mechanical ventilation induced lung and brain injury in mice, manifested in increased inflammatory factors in the bronchoalveolar lavage fluid and hippocampus, prolonged escape latency, and swimming distance and time in the target quadrant with a weakened concentration of neurons in the hippocampus. Our results presented elevated ATP and P2Y1R expressions in the mechanically ventilated mice and stretched MLE-12 cells. The mechanically ventilated mice and P2Y1 receptor activator MRS2365-treated HT-22 cells presented with elevated levels of DA and dysbindin-1. Inactivation of P2Y1 receptor in the hippocampus or blockage of DA receptor alleviated brain injury induced by mechanical ventilation in mice. To conclude, the current study elicited that lung injury induced by mechanical ventilation exacerbated brain injury in mice by increasing ATP production, activating the P2Y1 receptor, and thus promoting DA release.

## 1. Introduction

Mechanical ventilation is a resuscitative strategy for patients with critical illness, however, an inadvertent injury to the lungs could be induced during mechanical ventilation due to vigorous local alveolar overstretches and repetitive alveolar collapse, which is defined as ventilator-induced lung injury [1,2]. Essentially, insult due to mechanical ventilation is not restricted to the lungs individually but can span to the brain tissues, and the process is named lung-brain interaction [3]. Notably, combinations of experimental and preclinical models have demonstrated that lung injury induced by mechanical ventilation could progress to brain injury through numerous metabolic pathways, including nervous, hormonal, neuroendocrine, and inflammation regulation [4–6]. Meanwhile, brain injury can result in lung injury [7]. However, the underlying mechanism of lung-brain interaction remains elusive. It’s well-acknowledged that mechanical ventilation is detrimental to neurocognitive function [8]. Numerous studies have shown that mechanical ventilation can radically increase the concentration of hippocampal inflammatory cells by activation of the vagus nerve [9] and can affect postoperative memory dysfunction in mice [10]. According to existing reports, modifications of the internal environment (such as blood pressure, temperature, or pH value) activate visceral sensory neurons, which serve as transmitters of action potentials to the brainstem along the vagus nerve, and the nervous system maintains physiological homeostasis with the regulation of the reflex pathway of organ function [11]. Previously, the protective effect of bilateral vagotomy before mechanical ventilation has been proven on vector-induced brain injury in mice [12]. The aforementioned literature necessitates extensive investigation of the lung-brain interaction in brain injury induced by mechanical ventilation.

Extracellular adenosine triphosphate (ATP) is fundamental in ischemic injury to the central nervous system [13]. An existing study established an explicit correlation between the Transient receptor potential vanilloid 4 (TRPV4) channel and pannexin-1-mediated ATP production under stretch/strain conditions [14]. ATP release into the alveoli by activation of the purinergic receptors pulmonary vagal afferent neurons to stimulate vagal signaling can exacerbate the severity of an underlying brain injury [12]. ATP is implicated in acute lung inflammation induced by the P2Y receptor and lung injury induced by mechanical ventilation [15]. Additionally, P2Y receptor is implicated in the interaction of neurons and glial cells while vagal signaling can influence learning, memory, and locomotor behavior [16]. Moreover, overexpression of P2Y1 receptors can exacerbate the degree of neuronal injury evoked in traumatic brain injury [17]. Cognitive dysfunction after stimulation of the P2Y1 receptors is associated with short-term and long-term increased dopamine (DA) concentrations in the medial prefrontal cortex [18]. DA antagonism or blockage of DA receptors can effectively hinder hippocampal cell apoptosis induced by mechanical ventilation [19]. However, the interactions among ATP, P2Y1 receptor, and DA in lung-brain interaction remain elusive. In light of the aforementioned findings, we speculated that lung injury due to mechanical ventilation can reduce the degree of brain injury with activation of the ATP-mediated vagal signaling and P2Y1 receptor and stimulating DA release in the hippocampus. To verify the hypothesis, the current study sought to offer a theoretical basis for the management of brain injury induced by mechanical ventilation.

## 2. Materials and methods

### 2.1. Ethics statement

All animal experiments were monitored and conducted with the approval of the Ethical Committee of The First Affiliated Hospital of Zhengzhou University. Optimal measures were taken to reduce the number of animals and their suffering.

### 2.2. Reagents

MRS2365[(1 R,2 R,3S,4 R,5S)-4-[6-Amino-2-(methylthio)-9 H-pur-in-9-yl]-2,3-dihydroxybicyclo[3.1.0]hex-1-yl]methyl] diphosphoric acid mono ester trisodium salt (2157/1, Ellisville, MO, USA) and MRS2279 (N6-methyl-(N)-methanocarba-2’-deoxyadenosine-3’,5’-bisphosphate) provided by MedChemExpress LLC (HY-108657, Monmouth Junction, NJ, USA) were both dissolved in ACSF (artificial cerebral spinal fluid) (126 mM NaCl, 2.5 mM KCl, 1.2 mM NaH2PO4, 1.3 mM MgCl2, 2.4 mM CaCl2, pH 7.4).

### 2.3. Animals

C57BL6 mice aged 8–12 weeks provided by the Vital River Laboratory Animal Technology [SYXK(Beijing)2017–0033, Beijing, China] were housed in specific pathogen-free animal rooms with ad libitum access to food and water under 12/12 h light-dark cycles. The spontaneously breathing mice (sham group) received the same sedation as the mice in other groups: low-pressure ventilation (LVT group) [peak inspiratory pressure (PIP) of 12 cm H_2_O; positive end-expiratory pressure of 2 cm H_2_O; respiratory rate of 100 breaths/min] or high-pressure ventilation (HVT group) (PIP of 20 cm H_2_O; positive end-expiratory pressure of 0 cm H_2_O; respiratory rate of 50 breaths/min) for 90 min, followed by an array of 330-min long-term ventilation experiments under high-pressure ventilation [19]. High-pressure ventilated mice were randomly selected and intraperitoneally injected with the DA receptor antagonist haloperidol (0.5 mg/kg in 0.2 mL saline) 30 min prior to mechanical ventilation with the mice injected with an equivalent amount of normal saline as controls, or simultaneously intracerebroventricularly (coordinates with respect to bregma: AP = 0.4 mm; L = 0.95 mm) injected with 2 μL of the P2Y1 receptor antagonist MRS2279 (1 nmol, 96% purity; Tocris Bioscience, Abingdon, UK) 30 min prior to mechanical ventilation [20] with mice injected with an equivalent amount of artificial cerebrospinal fluid (ACSF) as controls. Animals were assigned into the following groups with 12 mice in each group (total 96): 1. the sham group, spontaneous breathing; 2. the LVT group, low tidal volume; 3. the HVT group, high tidal volume; 4. the long term group, mechanical ventilation for 330 min under high tidal volume; 5. the HVT + ACSF group, high tidal volume mechanical ventilation was performed 30 min after intracerebroventricular injection of ACSF; 6. the HVT + mrs2279 group, high tidal volume mechanical ventilation was performed 30 min after lateral ventricular injection of MRS2279; 7. the HVT + saline group, normal saline was injected intraperitoneally 30 min before mechanical ventilation; 8. the HVT + haloperidol group, haloperidol was injected intraperitoneally 30 min before mechanical ventilation. All ventilated mice were euthanized (intraperitoneal administration of 200 mg/kg pentobarbital sodium) after conducting the Morris water maze test. Hippocampus and lung tissues of mice were harvested for subsequent experimentation. The tissues were randomly selected from 6 mice in each group for pathological examination while the tissues of the remaining mice were homogenized for protein expression detection.

### 2.4. Morris water maze test

The Morris water maze test was conducted for an analysis of the spatial learning ability and reference memory of the mechanically ventilated mice [21]. The test depends on distal cues to navigate from the start point around the open swimming field to locate a submerged escape platform. All mice were trained for period of 1 week with assistance to find the hidden platform and mechanically ventilated on the 8^th^ day. Data including the escape latency was detected using the Anonymous tracking software (Stoelting Co., Wooddale, IL, USA).

### 2.5. BALF collection and protein detection

The lungs were rinsed using 0.8 mL sterile normal saline (0.9% NaC1, pre-heated) twice in situ, and the bronchoalveolar lavage fluid (BALF) was recovered and pooled, followed by centrifugation at 1000 g for 10 min for subsequent analysis [21]. The protein level in the BALF supernatant was detected using the bicinchoninic acid (BCA) kits (Beyotime, Shanghai, China).

### 2.6. Histopathological examination

The fixation of the left lower lobe of the lung was conducted by instillation of formalin into the airway at the pressure of 5 cm H_2_O and immersed in the same fixative. The tissue pathological scores were assessed by an experienced pathologist blind to this study after hematoxylin and eosin (H&E) staining. The fixed lung tissues were paraffin-embedded, sliced at 5 μm, dewaxed, hydrated, immersed in distilled water, stained with hematoxylin for 3 min, and differentiated with hydrochloric acid alcohol for 15 s. After a minor rinse, the slices were placed in the blue buffer for 15 s, rinsed under running water, stained with eosin for 3 min, rinsed, and fixed, followed by observation under the microscope. Parameters such as pulmonary edema, bleeding, white cell infiltration, alveolar septum thickening, and alveolar expansion of each mouse were evaluated using lung injury score (LIS) to verify the lung injury [22]. The total LIS was estimated with addition by adding the scores of each parameter (0–4) with a maximum score of 16.

After a rinse, the hippocampus was dehydrated using gradient ethanol (70%, 80%, and 90%), supplemented with an equivalent amount of a combination of pure alcohol and xylene for 15 min, and cleared with xylene I and xylene II (15 min each). This step was followed by the addition of an equal amount of a combination solution of xylene and paraffin for 15 min and paraffin I and paraffin II (50–60 min each). The tissues were paraffin-embedded, sectioned at 5 μm, baked, dewaxed, and hydrated. After a rinse under distilled water for 1 min, the sections were supplemented with 1% toluidine blue solution and stained for 25 min in a 54°C incubator. Subsequently, the sections were rinsed under distilled water, decolored with 95% ethanol for 30 s, cleared with xylene, and finally sealed. The number of Nissl-positive cells was observed under the microscope [23].

### 2.7. Enzyme-linked immunosorbent assay (ELISA)

The levels of several factors such as the tumor necrosis factor-α (TNF-α, MTA00B, R&D Systems Inc., Minneapolis, MN, USA), IL-1β (MLB00C, R&D Systems), interleukin (IL)-6 (M6000B, R&D Systems), and DA (Shanghai Yueyan Biological Technology, Shanghai, China) in the BALF and hippocampal tissue homogenate were detected using the commercially available ELISA kits [24].

### 2.8. Cell culture and treatment

Mouse hippocampal neuron cell line HT-22 and lung epithelial cell line MLE-12 (Chinese Academy of Sciences cell bank, Shanghai, China) were cultured in Dulbecco’s modified Eagle’s medium (Gibco Life Technologies, Carlsbad, CA, USA) or RPMI-1640 medium (Gibco) supplemented with a combination of 10% fetal bovine serum and 1% penicillin/streptomycin (100 µg/mL, Gibco) at 37°C with 95% air and 5% CO_2_. The HT-22 cells were supplemented with 1 µM of the P2Y1 receptor activator MRS2365 to activate the P2Y1 receptor (P2Y1R) in cells supplemented with an equivalent amount of ACSF as controls [25].

### 2.9. Cell stretch assay

Mouse lung epithelial cell line MLE-12 was exposed to 2.5 Hz or 1.0 Hz cyclic stretch with 5% or 18% elongation for 2 h in the FX-4000 T Flexcell T Dimension Plus system (Flexcell International, Burlington, NC, USA) in compliance with an earlier protocol [12].

### 2.10. ATP measurement

In accordance with an existing protocol [15], the ATP content in the BALF supernatant and supernatant of MLE-12 culture medium was measured by means of luciferase assay using the commercially available ATP detection kit (Beyotime). The relative light intensity was documented using the Fluoroskan luminometer (Thermo Fisher Scientific, Waltham, MA, USA).

### 2.11. Western blot

The total protein content was extracted from the tissues or cells using a combination of radio immunoprecipitation assay-buffer (Sigma-Aldrich, St. Louis, MO, USA) and quantified using BCA kits (Beyotime). Next, the protein sample was separated using 10% sodium dodecyl sulfate-polyacrylamide gel electrophoresis and transferred onto polyvinylidene fluoride membranes [22]. After membrane blockade using 5% skim milk, the membranes were incubated with the corresponding primary antibodies overnight at 4°C, followed by incubation with the secondary antibody IgG (ab205718, at a dilution ratio of 1:2000, Abcam, Cambridge, MA, USA). The protein band was developed using the enhanced chemiluminescence kit (Beyotime) while the gray value was analyzed using the NIH Image J software (NIH, Bethesda, MD, USA). The primary antibodies included P2Y1R (NBP1-69,246, at a dilution ratio of 1:1000, Novus Biologicals, Littleton, CO, USA), dysbindin-1 (PA5-27,371, at a dilution ratio of 1:1000, Thermo Fisher Scientific), and GAPDH (ab8245, at a dilution ratio of 1:2000, Abcam).

### 2.12. Statistical analysis

The SPSS21.0 statistical software (IBM Corp. Armonk, NY, USA) and GraphPad Prism 8.0 software (GraphPad Software, San Diego, CA, USA) were used for analysis and plot data. The measurement data were expressed as mean ± standard deviation. Normal distribution and variance homogeneity were firstly detected to conform to normal distribution and homogeneity of variance. Pairwise comparisons were analyzed using the *t* test and multi-group comparisons were analyzed using one-way or 2-way analysis of variance (ANOVA), followed by Tukey’s test. The *p* value was obtained from the bilateral tests. In all statistical references, a value of *p* < 0.05 was indicative of statistically significant differences, while *p* < 0.01 was indicative of an extremely significant statistical difference.

## 3. Results

To determine the role of mechanical ventilation in the interaction of lung-brain injury, we successfully established the lung injury model in mice by mechanical ventilation and evaluated the degree of lung injury and brain injury in mice. Our findings demonstrated that mechanical ventilation caused lung injury in mice, increased the level of ATP in the lung tissue, activated the P2Y1 receptor, which subsequently facilitated the release of dopamine in the mouse hippocampus, thus resulting in detrimental brain injury.

### 3.1. Mechanical ventilation induced lung injury in mice

**Figure 1.**
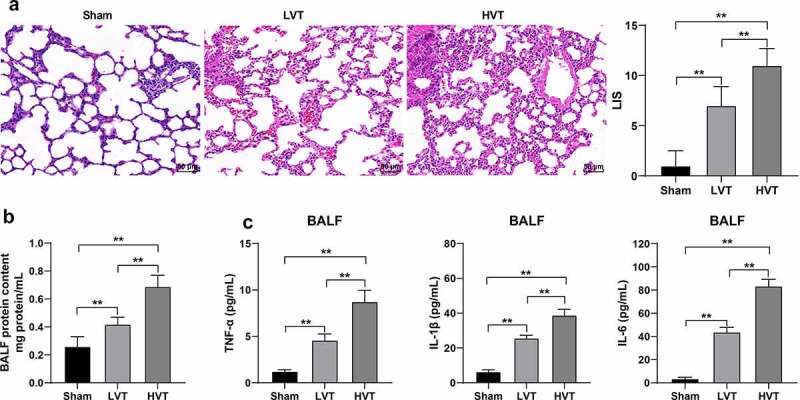
Mechanical ventilation induced lung injury in mice. The mice were mechanically ventilated, and the low tidal volume and high tidal volume were set. The mice with spontaneous breathing were used as the control. A: lung tissue injury in mice after mechanical ventilation observed by H&E staining and LIS scores; B: total protein level in mouse BALF detected by BCA method; C: levels of TNF-α, IL-1β, and IL-6 in mouse BALF detected by ELISA. N = 6. **p < 0.01. Data were analyzed using one-way ANOVA, followed by Tukey’s multiple comparisons test. LVT, low tidal volume; HVT, high tidal volume; LIS, lung injury score; BALF, bronchoalveolar lavage fluid; TNF-α, tumor necrosis factor-α; IL, interleukin.

To observe the mechanical ventilation-induced damage to lung tissues in mice, the mice were mechanically ventilated and the degree of lung tissue injury was observed by H&E staining. Mechanically ventilated mice presented with more discernible lung tissue injury relative to the spontaneously breathing mice, accompanied with evident pulmonary edema, inflammatory cell infiltration, alveolar septal thickening, and hyper-expansion (Figure 1a). Elevated LIS scores were identified with notable elevation in the HVT group relative to that in the LVT group (*p*< 0.01, Figure 1a). The total protein content in the BALF and levels of TNF-α, IL-1β, and IL-6 of mechanically ventilated mice were increased compared to the spontaneously breathing mice (*p*< 0.01, Figure 1b-c). The preceding results demonstrated that mechanical ventilation induced significant damage to the lung tissues in mice and the injury severity was associated with the ventilation volume.

### 3.2. Mechanical ventilation induced brain injury in mice

**Figure 2.**
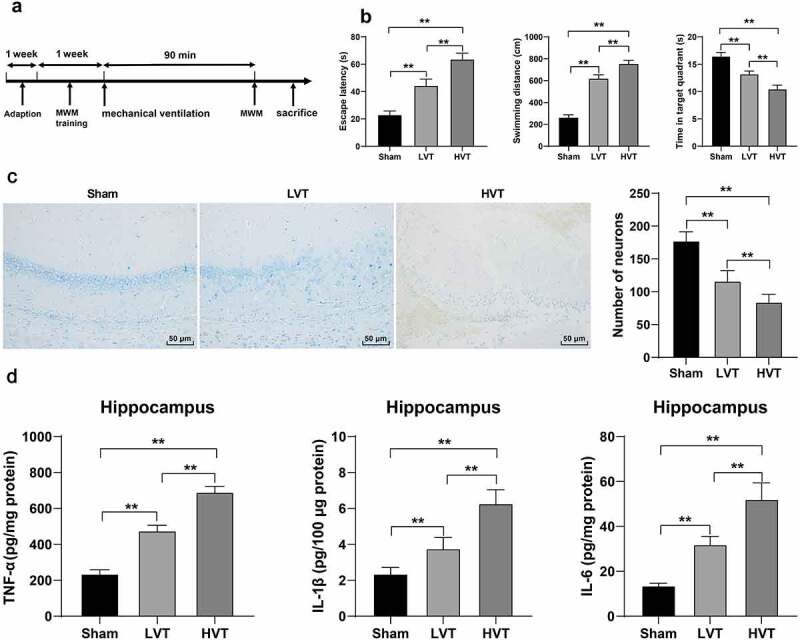
Mechanical ventilation induced brain injury in mice. The mice were mechanically ventilated, and the low tidal volume and high tidal volume were set. The mice with spontaneous breathing were used as the control. A: all mice were trained for Morris water maze test for 1 week before 90-min mechanical ventilation; B: cognitive function, including escape latency, swimming distance and time spent in target quadrant detected by Morris water maze test, N = 12; C: the number of neurons in mouse hippocampus observed by Nissl staining, N = 6; D: levels of TNF-α, IL-1β, and IL-6 in the hippocampus detected by ELISA, N = 6. **p < 0.01. Data were analyzed using one-way ANOVA, followed by Tukey’s multiple comparisons test. MWM, Morris water maze; LVT, low tidal volume; HVT, high tidal volume; TNF-α, tumor necrosis factor-α; IL, interleukin.

We subsequently observed the effect of mechanical ventilation on the cognitive function of mice by means of the Morris water maze test. The mice were trained for 1 week on the water maze test before mechanical ventilation and subsequent testing (Figure 2a). The experimental results exhibited that mechanical ventilation prolonged the escape latency (sham group: 23.04 ± 2.82 seconds; LVT group: 44.34 ± 4.85 seconds, HVT group: 63.61 ± 4.49 seconds), longer swimming distances (sham group: 264.18 ± 22.56 cm; LVT group: 620.61 ± 33.29 cm, HVT group: 756.53 ± 30.03 cm), and shortened time span in the target quadrant (sham group: 16.46 ± 0.72 seconds; LVT group: 13.21 ± 0.55 seconds, HVT group: 10.45 ± 0.72 seconds) in mice compared to the spontaneously breathing mice, with more notable alterations in the HVT group relative to the LVT group (*p*< 0.01, Figure 2b). Meanwhile, our findings presented a reduced number of neurons in the hippocampus of the mechanically ventilated mice (*p*< 0.01, Figure 2c), along with elevated secretion of inflammatory factors (TNF-α, IL-1β, and IL-6) in the hippocampus (*p*< 0.01, Figure 2d). The preceding results signified that mechanical ventilation induced lung injury along with brain injury in mice, where the injury severity was associated with the ventilation volume.

### 3.3. Mechanical ventilation increased alveolar free ATP and activated P2Y1 receptor

**Figure 3.**
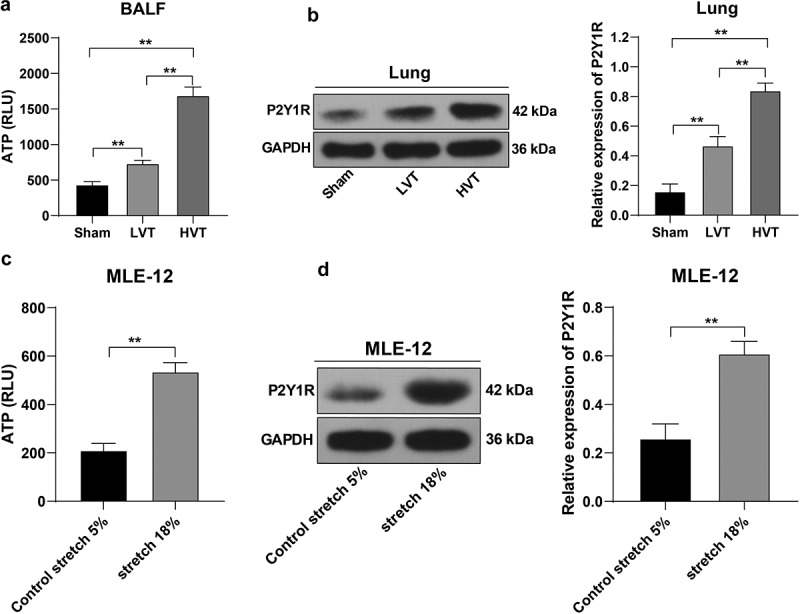
Mechanical ventilation increased alveolar free ATP and activated P2Y1 receptor. The mice were mechanically ventilated, and the low tidal volume and high tidal volume were set. The mice with spontaneous breathing were used as the control. A: ATP level in mouse BALF detected by luciferase assay, N = 6; B: PY1R expression pattern in lung tissues detected by Western blot, N = 6; C: ATP level in MLE-12 cells detected by luciferase assay; D: PY1R expression pattern in MLE-12 cells detected by Western blot. Cell experiments were conducted 3 times independently. **p < 0.01. Data in panels A and B were analyzed using one-way ANOVA, followed by Tukey’s multiple comparisons test, and data in panels C and D were analyzed using *t* test. ATP, adenosine triphosphate; RLU, relative light unit; BALF, bronchoalveolar lavage fluid; LVT, low tidal volume; HVT, high tidal volume; P2Y1R, P2Y1 receptor; GAPDH, glyceraldehyde-3-phosphate dehydrogenase.

Next, we detected the ATP content and P2Y1R expression pattern in mouse BALF and found an increased ATP content in BALF and elevated P2Y1R expression pattern in lung tissues after mechanical ventilation compared with the sham group, with notably higher levels in the HVT group than the LVT group (*p*< 0.01, Figure 3a-b). Simultaneously, the cell stretch assay verified alterations in ATP release and P2Y1R expression pattern in the stretched MLE-12 cells (*p*< 0.01, Figure 3c-d). Conjointly, our results elicited that mechanical ventilation stimulated ATP production and activated the P2Y1 receptor.

### 3.4. Activated P2Y1 receptor promoted DA release in the mouse hippocampus

**Figure 4.**
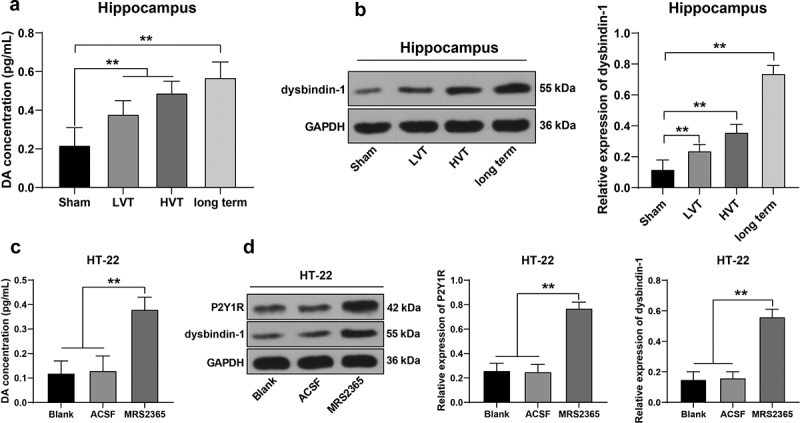
Activated P2Y1 receptor promoted DA release in mouse hippocampus. The mice were mechanically ventilated, and the low tidal volume and high tidal volume were set. The mice with spontaneous breathing were used as the control. Based on the high tidal volume, mice were performed with 330-min high positive-pressure ventilation. A: DA level in mouse hippocampus detected by ELISA, N = 6; B: dysbindin-1 level in mouse hippocampus detected by Western blot, N = 6; mouse hippocampal neuron cell line HT-22 were treated with P2Y1 receptor activator MRS2365 with cells treated with ACSF as controls, C: DA level in HT-22 cells detected by ELISA; D: levels of P2Y1R and dysbindin-1 in HT-22 cells detected by Western blot. Cell experiments were conducted 3 times independently. **p < 0.01. Data were analyzed using one-way ANOVA, followed by Tukey’s multiple comparisons test. DA, dopamine; LVT, low tidal volume; HVT, high tidal volume; GAPDH, glyceraldehyde-3-phosphate dehydrogenase; ACSF, artificial cerebral spinal fluid; P2Y1R, P2Y1 receptor.

Next, we determined whether mechanical ventilation results in cognitive dysfunction and brain injury in mice by stimulating the P2Y1 receptor and promoting DA release in the hippocampus. ELISA demonstrated an increase in the DA level in mechanically ventilated mice relative to the spontaneously breathing mice (*p*< 0.01, Figure 4a). The participation of protein dysbindin-1 is evident in the recycling of DRD2 where it antagonizes the effect of DA in the high dopaminergic state as a compensatory mechanism [19]. Our results revealed that the dysbindin-1 protein was manipulated after mechanical ventilation (*p*< 0.01, Figure 4b). To further validate the relationship between ventilation time and dopamine secretion, the experimental mice were subject to ventilation for an extended period of 330 min under high positive-pressure ventilation, with an observation of notable increases in the DA level and dysbindin-1 protein (*p*< 0.01, Figure 4a-b). Altogether, our findings elicited that continuous mechanical ventilation increased the DA level in the mouse hippocampus.

To further verify the association of DA release with P2Y1 receptor activation, the mouse hippocampal neurons HT-22 were only treated with the P2Y1 receptor activator MRS2365 *in vitro* to activate the P2Y1R expression pattern (*p*< 0.01, Figure 4d). The levels of DA and dysbindin-1 protein were significantly elevated in the HT-22 cells (*p*< 0.01, Figure 4c-d). These results elucidated that P2Y1 receptor activation had notably facilitated DA release in the mouse hippocampus.

### 3.5. Inhibition of P2Y1 receptor activation ameliorated brain injury induced by mechanical ventilation in mice

**Figure 5.**
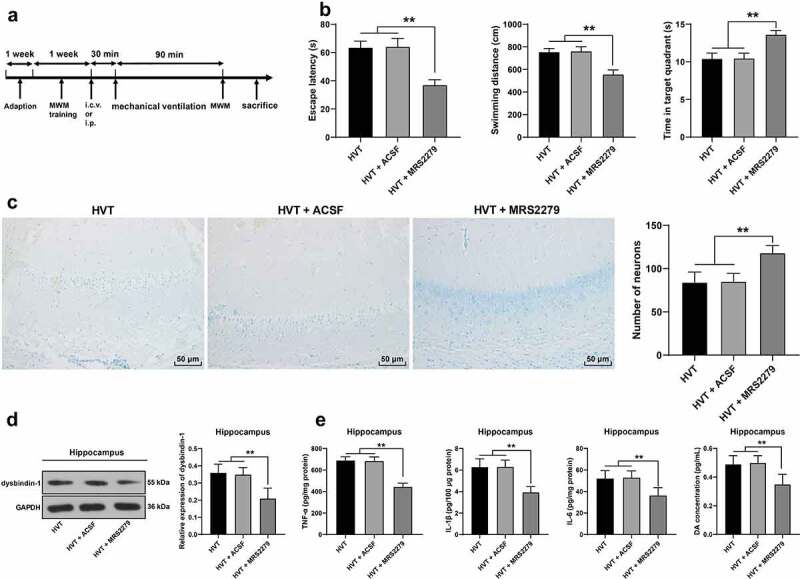
Inhibition of P2Y1 receptor activation ameliorated brain injury induced by mechanical ventilation in mice. A: mice were intracerebroventricularly injected with P2Y1 receptor antagonist MRS2279 30 min before mechanical ventilation, with the injection of ACSF as the control; B: cognitive function, including escape latency, swimming distance, and time spent in the target quadrant was detected by Morris water maze test, N = 12; C: the number of neurons in mouse hippocampus was observed by Nissl staining, N = 6; D: dysbindin-1 level was detected by Western blot, N = 6; E: levels of TNF-α, IL-1β, and IL-6 and DA in hippocampus were detected by ELISA, N = 6. **p < 0.01. Data were analyzed using one-way ANOVA, followed by Tukey’s multiple comparisons test. MWM, Morris water maze; LVT, low tidal volume; HVT, high tidal volume; ACSF, artificial cerebral spinal fluid; GAPDH, glyceraldehyde-3-phosphate dehydrogenase; TNF-α, tumor necrosis factor-α; IL, interleukin; DA, dopamine.

To validate whether mechanical ventilation induced brain injury by activating P2Y1 receptor in the mouse hippocampus, the experimental mice were intracerebroventricularly injected with the P2Y1 antagonist MRS2279 30 min prior to mechanical ventilation, using an injection of ACSF as control (Figure 5a). Mice exhibited shorter latency (HVT group: 63.61 ± 4.49 seconds; HVT + ACSF group: 64.25 ± 5.81 seconds, HVT + MRS2279 group: 37.17 ± 3.50 seconds) and swimming distances (HVT group: 756.53 ± 30.03 cm; HVT + ACSF group: 762.83 ± 38.06 cm, HVT + MRS2279 group: 559.0 ± 37.63 cm) and spent a longer period of time in the target quadrant (HVT group: 10.45 ± 0.72 seconds; HVT + ACSF group: 10.48 ± 0.67 seconds, HVT + MRS2279 group: 13.63 ± 0.54 seconds) after inhibiting P2Y1 receptor activation (*p*< 0.05, Figure 5b). Meanwhile, the number of neurons had increased (*p*< 0.05, Figure 5c), while the dysbindin-1 protein level was decreased in the hippocampus (*p*< 0.05, Figure 5d), and levels of TNF-α, IL-1β, IL-6, and DA were all decreased in the hippocampus (*p*< 0.05, Figure 5e). Overall, our findings elicited that inhibition of P2Y1 receptor activation attenuated mouse brain injury induced by mechanical ventilation.

### 3.6. Blockage of DA receptor mitigated mouse brain injury induced by increased DA release mediated by mechanical ventilation via P2Y1 receptor activation

**Figure 6.**
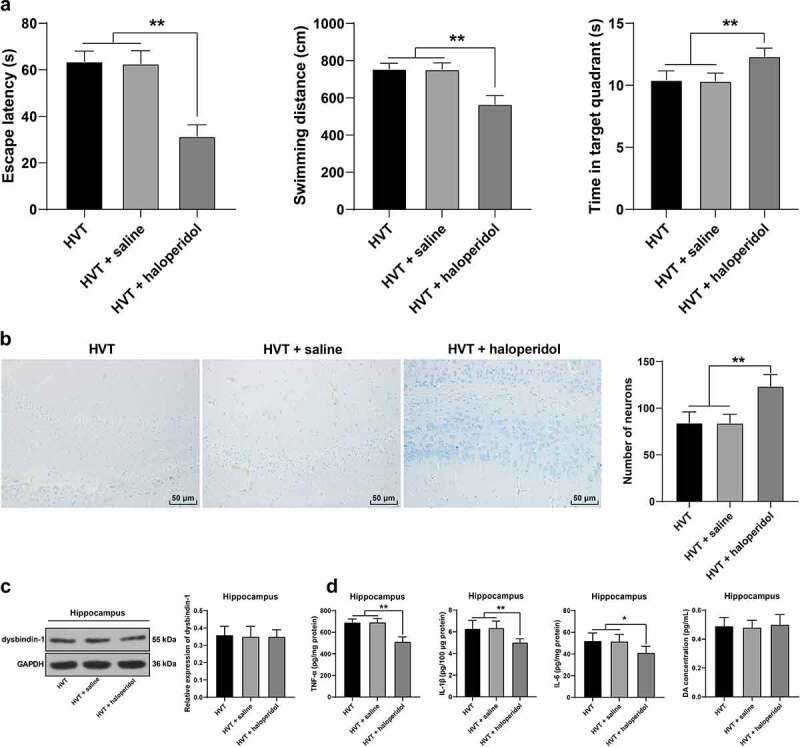
Blockage of DA receptor mitigated mouse brain injury induced by increased DA release mediated by mechanical ventilation via P2Y1 receptor activation. Mice were intraperitoneally injected with haloperidol 30 min before mechanical ventilation, with the injection of saline as the control. A: cognitive function, including escape latency, swimming distance, and time spent in the target quadrant was detected by Morris water maze test, N = 12; B: the number of neurons in mouse hippocampus was observed by Nissl staining, N = 6; C: dysbindin-1 level was detected by Western blot, N = 6; D: levels of TNF-α, IL-1β, and IL-6 and DA in hippocampus were detected by ELISA, N = 6. *p < 0.05, **p < 0.01. Data were analyzed using one-way ANOVA, followed by Tukey’s multiple comparisons test. LVT, low tidal volume; HVT, high tidal volume; GAPDH, glyceraldehyde-3-phosphate dehydrogenase; TNF-α, tumor necrosis factor-α; IL, interleukin; DA, dopamine.

Similarly, to validate whether P2Y1 receptor activation induced brain injury DA nerve conduction by increasing the DA expression pattern, the experimental mice were intraperitoneally injected with the DA receptor antagonist haloperidol 30 min before mechanical ventilation (Figure 5a). Similar to the function of MRS2279, the mice injected with haloperidol showed vital improvements in cognition after mechanical ventilation (*p*< 0.05, Figure 6a), with shorter latency (HVT group: 63.61 ± 4.49 seconds; HVT + saline group: 62.71 ± 5.54 seconds, HVT + haloperidol group: 31.53 ± 4.82 seconds) and swimming distances (HVT group: 756.53 ± 30.03 cm; HVT + saline group: 753.46 ± 34.13 cm, HVT + haloperidol group: 565.85 ± 45.98 cm) and spent a longer period of time in the target quadrant (HVT group: 10.45 ± 0.72 seconds; HVT + saline group: 10.36 ± 0.63 seconds, HVT + haloperidol group: 12.34 ± 0.67 seconds). Meanwhile, the number of neurons had increased in the hippocampus (*p*< 0.05, Figure 6b), the levels of DA and dysbindin-1 protein showed no significant alterations (*p*> 0.05, Figure 6c-d), while the levels of TNF-α, IL-1β, and IL-6 were reduced (*p*< 0.05, Figure 6d). Collectively, our findings suggested that suppression of the DA receptor mitigated mouse brain injury induced by increased DA release mediated by mechanical ventilation via P2Y1 receptor activation.

## 4. Discussion

**Figure 7.**
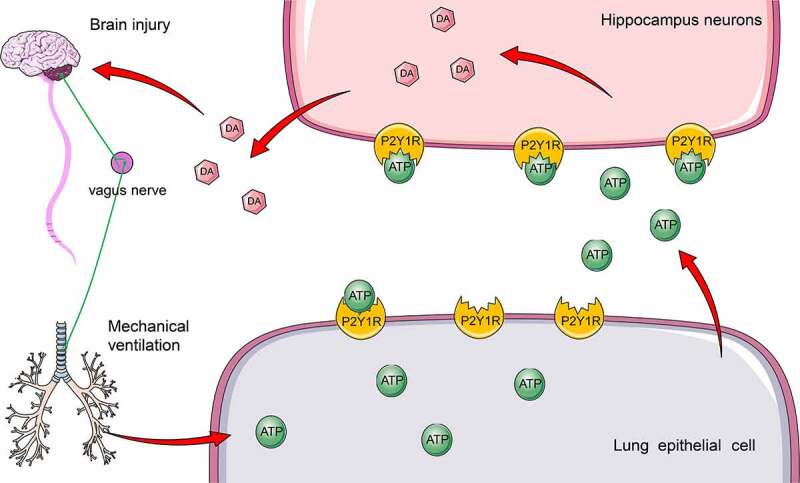
Lung injury induced by mechanical ventilation increased ATP production, activated P2Y1 receptor, and promoted DA release in the hippocampus, and thus exacerbated brain injury.

Ventilator-induced lung injury can exacerbate the severity of systemic inflammatory response that ultimately progresses to extra-pulmonary organ failure [26]. The current study revealed that lung injury induced by mechanical ventilation primitively increased ATP production, activated the P2Y1 receptor, and promoted DA release in the hippocampus, thus resulting in an exacerbated brain injury (Figure 7).

Improper mechanical ventilation can lead to an acute injury to the lungs [27]. To observe the lung tissue insult induced by mechanical ventilation, the experimental mice were mechanically ventilated. Our results demonstrated the presence of alveolar edema, inflammatory infiltration, thickened alveolar septum as well as alveolar over-expansion upon mechanical ventilation. Mechanical ventilation can initiate the production of terminal inflammatory factors IL-6 and TNF-α in the lung [28]. Our results presented definitive elevations in the levels of TNF-α, IL-1β, and IL-6 in mechanically ventilated mice relative to the spontaneously breathing mice. Previously, an existing study identified lung injury in rats induced due to short-term mechanical ventilation with hyperoxia [29]. Consistently, our results demonstrated lung injury resultant of mechanical ventilation in mice. Additionally, ventilation can harm the central nervous system via pulmonary inflammation [30]. We herein examined the cognitive function of mice with mechanical ventilation and observed that the mechanically ventilated mice exhibited longer escape latency and swimming distance and a shorter period of time in the target quadrant than spontaneously breathing mice. Simultaneously, our results indicated that the mechanically ventilated mice had a decreased number of neurons and increased levels of TNF-α, IL-1β, and IL-6 in the hippocampus. An existing study identified ventilator-induced lung injury as an exacerbating factor or trigger of brain injury [31]. Additionally, mechanical ventilation presents with an elevated risk for cerebral inflammation and brain injury [32]. Altogether, our findings indicated that mechanical ventilation can precipitate brain injury in mice.

Excessive extracellular ATP concentration can facilitate the degree of ventilator-induced lung injury by desensitizing the P2Y2 and P2X4 receptors [33]. Purinergic receptors can fundamentally improve pro-inflammatory cytokine responses in lung infection [34]. Therefore, an analysis of the alterations in the ATP and P2Y1 receptors presented with an elevated ATP content and P2Y1R expression in the mechanically ventilated mice with stretched MLE-12 cells. An existing study determined the capacity of mechanical ventilation to increase extracellular ATP release and exacerbate the degree of pulmonary edema, inflammation, and lung injury [35]. P2Y1R plays a critical role in ATP-induced ventilation elevation [36]. Collectively, our findings elicited that mechanical ventilation stimulated ATP production and activated P2Y1R.

P2Y1 receptor inhibition can restore impaired synaptic plasticity in the epileptic hippocampus [37]. P2Y1R can influence DA release in the cortical area in conditions of cognitive impairment [18]. We herein speculated that mechanical ventilation increases DA release by activation of the P2Y1 receptor and thus exacerbates the severity of cognitive dysfunction and brain injury. In the mechanically ventilated mice, we observed increased levels of DA and dysbindin-1. Dysbindin-1 was evident with a vital function in the maintenance of mesolimbic DA tone [38]. Our results illustrated that prolonged mechanical ventilation significantly increased the levels of DA and dysbindin-1 in mice. To verify whether P2Y1 receptor activation is implicated in DA release, the mouse hippocampal neuronal cells HT-22 were treated with the P2Y1 receptor activator MRS2365. Subsequent observation elicited a notable increase in the levels of DA and dysbindin-1 in the HT-22 cells. Extracellular ATP promotes DA release by activation of the P2 receptors dopaminergic system of the rat brain [39]. Consistently, an existing study identified the ability of P2Y1 receptor activation to facilitate DA release in the mouse hippocampus. Additionally, P2Y1R can manipulate neuronal and glial functions [40]. To determine the effect of P2Y1 receptor on ventilator-induced brain injury, the experimental mice were administered with the P2Y1R antagonist MRS2279 prior to mechanical ventilation. Our results indicated that mice treated with MRS2279 showed shorter escape latency and swimming distance as well as a prolonged time duration in the target quadrant in the Morris water maze test after mechanical ventilation. Additionally, the number of neurons had increased while the levels of DA and dysbindin-1 were decreased in the hippocampus. P2Y12Rs increase systemic vascular inflammation [41]. Our results showed that the P2Y1R antagonist significantly decreased the levels of TNF-α, IL-1β, and IL-6. An existing study identified that P2Y1R blockade could remarkably mitigate hippocampal neuronal death in rats [42]. Consistently, our findings elicited that suppression of the P2Y1 receptor activation had definitively alleviated brain injury induced by mechanical ventilation in mice.

As aforementioned, P2Y1 receptor activation can facilitate DA release. To investigate the role of DA in mechanical ventilation-induced brain injury, the experimental mice were treated with DA receptor antagonist haloperidol before conducting mechanical ventilation. Haloperidol has extensive clinical application for agitation in traumatic brain injury [43]. The mice treated with haloperidol exhibited significant improvement in cognitive function after mechanical ventilation. The antagonism of DA receptor D3 can terminally attenuate neuroinflammation in the mouse model of Parkinson’s disease [44]. Our results demonstrated that haloperidol pre-treatment reduced the levels of TNF-α, IL-1β, and IL-6 in mechanically ventilated mice. Knockdown of the DA D2 receptor comprehensively improves functional brain activity in rats [45]. Consistently, our findings elicited that inhibition of DA receptor ameliorated brain injury induced by mechanical ventilation.

## 5. Conclusions

To conclude, our results elicited that mechanical ventilation induced brain injury in mice by facilitating ATP production in the lungs, activating the P2Y1 receptor and increasing DA release. Although the current study revealed that mechanical ventilation could exacerbate brain injury in mice, the ventilation time in animal experiments presented with significant variations from the mechanical ventilation time in clinical application, which warrants extensive verification in clinical trials. Meanwhile, the underlying mechanism on how P2Y1 receptor activation signaling in the lung tissues is transmitted in the hippocampus and regulates DA release needs further investigation and analysis at the clinical level. Currently, limited studies have investigated the interaction between lung and brain caused by mechanical ventilation, and few preclinical studies on mouse modeling. Therefore, certain limitations are preliminary during the integration of the research results into clinical application. Our future studies conduct extensive investigations on the role of the vagus nerve in lung-brain interaction.

## Data Availability

The data that support this study are available from the corresponding author upon reasonable request.

## Research highlights

1. Mechanical ventilation induces lung injury and brain injury in mice.
2. Mechanical ventilation increases alveolar free ATP level.
3. Increased ATP levels activate P2Y1 receptor.
4. P2Y1 receptor activation promotes DA release in the hippocampus.
5. Mechanical ventilation promotes brain injury by facilitating DA release.
